# *N*-Trityl-2-(tritylsulfan­yl)aniline

**DOI:** 10.1107/S1600536811039183

**Published:** 2011-10-08

**Authors:** Adam Neuba, Ulrich Flörke, Gerald Henkel

**Affiliations:** a Department Chemie, Fakultät für Naturwissenschaften, Universität Paderborn, Warburgerstrasse 100, D-33098 Paderborn, Germany

## Abstract

The title compound, C_44_H_35_NS, is a derivative of amino­thio­phenol and possesses a protected *S*-triphenyl­methyl thio­ether and an *N*-triphenyl­methyl­amine functional group. The trityl groups show an *anti* orientation, with C—C—N—C and C—C—S—C torsion angles of −151.0 (3) and −105.3 (2)°, respectively. There is an intra­molecular N—H⋯S hydrogen bond.

## Related literature

For the synthesis, see: Neuba *et al.* (2011 ▶). For a related structure, see: Neuba *et al.* (2007 ▶). For background to complexes of amine guanidine hybrides with first row transition elements, see: Börner *et al.* (2009 ▶); Herres *et al.* (2005 ▶); Herres-Pawlis *et al.* (2005 ▶, 2009 ▶); Neuba *et al.* (2007 ▶, 2008 ▶, 2010 ▶, 2011 ▶); Pohl *et al.* (2000 ▶); Wittmann *et al.* (2001 ▶).
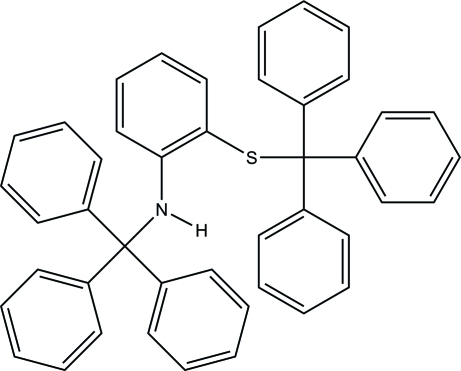


## Experimental

### 

#### Crystal data

C_44_H_35_NS
*M* *_r_* = 609.79
Monoclinic, 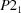

*a* = 8.2377 (10) Å
*b* = 23.513 (3) Å
*c* = 9.0894 (10) Å
β = 113.512 (3)°
*V* = 1614.4 (3) Å^3^
*Z* = 2
Mo *K*α radiation
μ = 0.13 mm^−1^
*T* = 120 K
0.36 × 0.23 × 0.20 mm

#### Data collection

Bruker SMART APEX diffractometer
Absorption correction: multi-scan (*SADABS*; Sheldrick, 2004 ▶) *T*_min_ = 0.953, *T*_max_ = 0.974
13465 measured reflections
7059 independent reflections
5106 reflections with *I* > 2σ(*I*)
*R*_int_ = 0.086

#### Refinement

*R*[*F* ^2^ > 2σ(*F* ^2^)] = 0.058
*wR*(*F* ^2^) = 0.099
*S* = 0.91
7059 reflections
419 parameters
1 restraint
H atoms treated by a mixture of independent and constrained refinement
Δρ_max_ = 0.36 e Å^−3^
Δρ_min_ = −0.33 e Å^−3^
Absolute structure: Flack (1983 ▶), 3130 Friedel pairs
Flack parameter: 0.03 (7)

### 

Data collection: *SMART* (Bruker, 2002 ▶); cell refinement: *SAINT* (Bruker, 2002 ▶); data reduction: *SAINT*; program(s) used to solve structure: *SHELXTL* (Sheldrick, 2008 ▶); program(s) used to refine structure: *SHELXTL*; molecular graphics: *SHELXTL*; software used to prepare material for publication: *SHELXTL* and local programs.

## Supplementary Material

Crystal structure: contains datablock(s) I, global. DOI: 10.1107/S1600536811039183/nk2100sup1.cif

Structure factors: contains datablock(s) I. DOI: 10.1107/S1600536811039183/nk2100Isup2.hkl

Supplementary material file. DOI: 10.1107/S1600536811039183/nk2100Isup3.cml

Additional supplementary materials:  crystallographic information; 3D view; checkCIF report

## Figures and Tables

**Table 1:** Hydrogen-bond geometry (Å, °)

*D*—H⋯*A*	*D*—H	H⋯*A*	*D*⋯*A*	*D*—H⋯*A*
N1—H1⋯S1	0.81 (3)	2.46 (3)	2.984 (3)	123 (1)

**Fig. 1.**
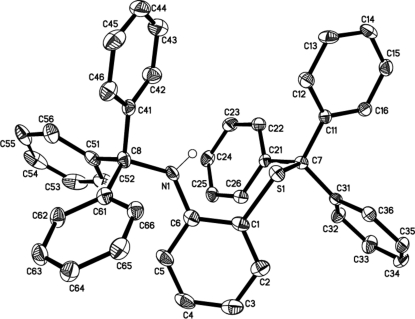
Molecular structure with displacement ellipsoids drawn at the 50% probability level. Aromatic-H atoms omitted for clarity.

## supplementary crystallographic information

### Comment

The synthesis and characterization of novel molecules containing nitrogen and sulfur as donor functions and their application in synthesis of sulfur copper complexes is important for biomimetic copper-sulfur chemistry. In search of multifunctional ligands we have extended our studies to guanidyl-type systems with N-donor functions. The first derivative, the ligand bis(tetramethyl-guanidino)propylene as well as amine guanidine hybrids and their complexes with Cu, Fe, Ni, Ag, Mn, Co and Zn have recently been investigated (Pohl *et al.*, 2000; Wittmann *et al.*, 2001; Herres-Pawlis *et al.*, 2005, 2009; Herres *et al.*, 2005; Neuba *et al.*, 2008; 2010; Börner *et al.* 2009). We have now developed several sulfur guanidine hybrids based on aminothiophenol and cysteamine (Neuba *et al.*, 2007; Neuba *et al.* 2011). The synthesized sulfur guanidine compounds possess aliphatic and aromatic thioethers or disulfide groups and were used in the synthesis of copper thiolate complexes to mimic active centres like the Cu_A_ in cytochrome-c oxidase and N_2_O-reductase. In the course of these experiments we synthesized the title compound as a by-product from the reaction of 1,1,3,3-tetramethyl-2-(2-(tritylthio)phenyl)guanidine with [Cu(MeCN)_4_]PF_6_ in MeCN (Neuba *et al.*, 2011). The molecular structure of C_44_H_35_NS shows two trityl units with dihedral angles between the phenyl planes C11—C16, C21—C26 and C31—C36 of 84.7 (1)°, 68.2 (1)° and 80.9 (1)° and for planes C41—C46, C51—C56 and C61—C66 the angles measure 88.7 (1)°, 77.6 (1)° and 74.5 (1)°, respectively. Orientation of the trityl groups relative to the C1—C6 ring is indicated from the torsion angles C1—C6—N1—C8 of 151.0 (3)° and C6—C1—S1—C7 of 105.3 (2)°. The title compound shows an intramolecular N—H···S bond with H···S 2.46 Å and N—H···S angle of 122°.

### Experimental

Full synthetic details and spectroscopic as well as analytical characterization is given in Neuba *et al.* (2011).

### Refinement

H atoms were clearly identified in difference syntheses, idealized and refined riding on the carbon atoms with C—H = 0.95 Å, and with isotropic displacement parameters *U*_iso_(H) = 1.2U(C/N_eq_). The N-bound H atom was refined freely.  The value of the Flack parameter (0.03 (7)) was determined from 3130 Friedel pairs.

### Crystal data

**Table:**

C_44_H_35_NS	*F*(000) = 644
*M**_r_* = 609.79	*D*_x_ = 1.254 Mg m^−^^3^
Monoclinic, *P*2_1_	Mo *K*α radiation, λ = 0.71073 Å
Hall symbol: P 2yb	Cell parameters from 756 reflections
*a* = 8.2377 (10) Å	θ = 2.6–25.0°
*b* = 23.513 (3) Å	µ = 0.13 mm^−^^1^
*c* = 9.0894 (10) Å	*T* = 120 K
β = 113.512 (3)°	Prism, colourless
*V* = 1614.4 (3) Å^3^	0.36 × 0.23 × 0.20 mm
*Z* = 2	

### Data collection

**Table:**

Bruker SMART APEX diffractometer	7059 independent reflections
Radiation source: sealed tube	5106 reflections with *I* > 2σ(*I*)
graphite	*R*_int_ = 0.086
φ and ω scans	θ_max_ = 27.9°, θ_min_ = 1.7°
Absorption correction: multi-scan (*SADABS*; Sheldrick, 2004)	*h* = −10→10
*T*_min_ = 0.953, *T*_max_ = 0.974	*k* = −30→30
13465 measured reflections	*l* = −11→9

### Refinement

**Table:**

Refinement on *F*^2^	Secondary atom site location: difference Fourier map
Least-squares matrix: full	Hydrogen site location: difference Fourier map
*R*[*F*^2^ > 2σ(*F*^2^)] = 0.058	H atoms treated by a mixture of independent and constrained refinement
*wR*(*F*^2^) = 0.099	*w* = 1/[σ^2^(*F*_o_^2^) + (0.0245*P*)^2^] where *P* = (*F*_o_^2^ + 2*F*_c_^2^)/3
*S* = 0.91	(Δ/σ)_max_ < 0.001
7059 reflections	Δρ_max_ = 0.36 e Å^−^^3^
419 parameters	Δρ_min_ = −0.33 e Å^−^^3^
1 restraint	Absolute structure: Flack (1983), 3130 Friedel pairs
Primary atom site location: structure-invariant direct methods	Flack parameter: 0.03 (7)

### Special details

**Table:**

Geometry. All e.s.d.'s (except the e.s.d. in the dihedral angle between two l.s. planes) are estimated using the full covariance matrix. The cell e.s.d.'s are taken into account individually in the estimation of e.s.d.'s in distances, angles and torsion angles; correlations between e.s.d.'s in cell parameters are only used when they are defined by crystal symmetry. An approximate (isotropic) treatment of cell e.s.d.'s is used for estimating e.s.d.'s involving l.s. planes.
Refinement. Refinement of *F*^2^ against ALL reflections. The weighted *R*-factor *wR* and goodness of fit *S* are based on *F*^2^, conventional *R*-factors *R* are based on *F*, with *F* set to zero for negative *F*^2^. The threshold expression of *F*^2^ > σ(*F*^2^) is used only for calculating *R*-factors(gt) *etc*. and is not relevant to the choice of reflections for refinement. *R*-factors based on *F*^2^ are statistically about twice as large as those based on *F*, and *R*- factors based on ALL data will be even larger.

### Fractional atomic coordinates and isotropic or equivalent isotropic displacement parameters (Å^2^)

**Table:**

	*x*	*y*	*z*	*U*_iso_*/*U*_eq_	
S1	0.84857 (9)	0.45253 (3)	0.53668 (8)	0.02420 (17)	
N1	0.8060 (3)	0.53874 (10)	0.7596 (3)	0.0266 (6)	
C1	1.0204 (4)	0.47875 (11)	0.7141 (3)	0.0254 (7)	
C2	1.1929 (4)	0.46002 (13)	0.7572 (4)	0.0309 (7)	
H2A	1.2192	0.4334	0.6911	0.037*	
C3	1.3274 (4)	0.47992 (13)	0.8964 (4)	0.0380 (9)	
H3A	1.4455	0.4669	0.9268	0.046*	
C4	1.2869 (4)	0.51873 (13)	0.9892 (4)	0.0384 (9)	
H4A	1.3777	0.5318	1.0859	0.046*	
C5	1.1180 (4)	0.53900 (12)	0.9452 (4)	0.0329 (8)	
H5A	1.0947	0.5665	1.0109	0.039*	
C6	0.9796 (4)	0.52008 (11)	0.8058 (3)	0.0253 (7)	
C7	0.7929 (3)	0.37851 (11)	0.5851 (3)	0.0183 (6)	
C8	0.7459 (4)	0.59533 (11)	0.7893 (3)	0.0240 (7)	
C11	0.6271 (3)	0.36367 (11)	0.4338 (3)	0.0199 (6)	
C12	0.4949 (4)	0.40342 (12)	0.3586 (4)	0.0294 (7)	
H12A	0.5070	0.4410	0.4000	0.035*	
C13	0.3454 (4)	0.38895 (13)	0.2239 (4)	0.0322 (8)	
H13A	0.2580	0.4170	0.1727	0.039*	
C14	0.3224 (4)	0.33499 (13)	0.1644 (3)	0.0294 (7)	
H14A	0.2195	0.3253	0.0726	0.035*	
C15	0.4505 (4)	0.29457 (13)	0.2391 (4)	0.0306 (7)	
H15A	0.4350	0.2568	0.1988	0.037*	
C16	0.6020 (4)	0.30866 (12)	0.3729 (3)	0.0241 (6)	
H16A	0.6891	0.2804	0.4231	0.029*	
C21	0.7538 (4)	0.37820 (11)	0.7363 (3)	0.0181 (6)	
C22	0.5905 (4)	0.36177 (13)	0.7314 (4)	0.0293 (7)	
H22A	0.4983	0.3527	0.6310	0.035*	
C23	0.5580 (4)	0.35815 (14)	0.8702 (4)	0.0337 (8)	
H23A	0.4450	0.3464	0.8635	0.040*	
C24	0.6880 (4)	0.37143 (13)	1.0161 (4)	0.0301 (7)	
H24A	0.6655	0.3695	1.1106	0.036*	
C25	0.8527 (4)	0.38774 (12)	1.0241 (3)	0.0267 (7)	
H25A	0.9436	0.3972	1.1249	0.032*	
C26	0.8859 (4)	0.39040 (11)	0.8872 (3)	0.0233 (6)	
H26A	1.0008	0.4007	0.8955	0.028*	
C31	0.9462 (3)	0.33825 (11)	0.5998 (3)	0.0194 (6)	
C32	1.0006 (4)	0.29479 (12)	0.7134 (3)	0.0266 (7)	
H32A	0.9472	0.2910	0.7882	0.032*	
C33	1.1315 (4)	0.25724 (14)	0.7182 (4)	0.0355 (8)	
H33A	1.1680	0.2282	0.7974	0.043*	
C34	1.2093 (4)	0.26109 (13)	0.6115 (4)	0.0322 (8)	
H34A	1.3001	0.2352	0.6170	0.039*	
C35	1.1552 (4)	0.30282 (13)	0.4955 (3)	0.0291 (7)	
H35A	1.2070	0.3053	0.4192	0.035*	
C36	1.0258 (4)	0.34111 (12)	0.4900 (3)	0.0242 (6)	
H36A	0.9902	0.3699	0.4100	0.029*	
C41	0.5692 (4)	0.60614 (12)	0.6443 (3)	0.0242 (7)	
C42	0.4388 (4)	0.56465 (13)	0.6016 (4)	0.0348 (8)	
H42A	0.4593	0.5305	0.6623	0.042*	
C43	0.2795 (4)	0.57226 (15)	0.4718 (4)	0.0442 (9)	
H43A	0.1917	0.5434	0.4435	0.053*	
C44	0.2479 (4)	0.62216 (16)	0.3829 (4)	0.0436 (9)	
H44A	0.1395	0.6273	0.2924	0.052*	
C45	0.3742 (4)	0.66375 (15)	0.4266 (4)	0.0389 (8)	
H45A	0.3521	0.6981	0.3669	0.047*	
C46	0.5341 (4)	0.65648 (13)	0.5570 (4)	0.0307 (7)	
H46A	0.6200	0.6860	0.5866	0.037*	
C51	0.7100 (4)	0.59382 (12)	0.9423 (3)	0.0244 (7)	
C52	0.7844 (4)	0.55322 (13)	1.0613 (4)	0.0355 (8)	
H52A	0.8529	0.5233	1.0448	0.043*	
C53	0.7604 (4)	0.55555 (16)	1.2037 (4)	0.0452 (10)	
H53A	0.8146	0.5277	1.2842	0.054*	
C54	0.6586 (5)	0.59792 (17)	1.2298 (4)	0.0460 (10)	
H54A	0.6456	0.6001	1.3289	0.055*	
C55	0.5768 (4)	0.63677 (15)	1.1108 (4)	0.0382 (8)	
H55A	0.5029	0.6653	1.1255	0.046*	
C56	0.6017 (4)	0.63455 (13)	0.9692 (4)	0.0321 (7)	
H56A	0.5433	0.6616	0.8876	0.038*	
C61	0.8871 (4)	0.64051 (11)	0.8003 (3)	0.0242 (7)	
C62	0.9465 (4)	0.68078 (12)	0.9228 (4)	0.0295 (7)	
H62A	0.8978	0.6819	1.0014	0.035*	
C63	1.0779 (4)	0.71948 (13)	0.9296 (4)	0.0364 (8)	
H63A	1.1179	0.7468	1.0135	0.044*	
C64	1.1499 (4)	0.71877 (13)	0.8180 (4)	0.0338 (8)	
H64A	1.2393	0.7453	0.8240	0.041*	
C65	1.0909 (4)	0.67883 (13)	0.6957 (4)	0.0324 (7)	
H65A	1.1407	0.6777	0.6177	0.039*	
C66	0.9601 (4)	0.64067 (12)	0.6869 (3)	0.0279 (7)	
H66A	0.9194	0.6140	0.6015	0.033*	
H1	0.742 (3)	0.5239 (12)	0.676 (3)	0.017 (8)*	

### Atomic displacement parameters (Å^2^)

**Table:**

	*U*^11^	*U*^22^	*U*^33^	*U*^12^	*U*^13^	*U*^23^
S1	0.0295 (4)	0.0185 (3)	0.0221 (4)	−0.0008 (3)	0.0077 (3)	0.0032 (3)
N1	0.0254 (14)	0.0186 (13)	0.0276 (15)	−0.0025 (11)	0.0018 (12)	−0.0080 (11)
C1	0.0235 (15)	0.0218 (15)	0.0280 (17)	−0.0080 (12)	0.0071 (13)	0.0032 (13)
C2	0.0270 (15)	0.0234 (16)	0.0445 (18)	−0.0023 (13)	0.0166 (14)	0.0061 (15)
C3	0.0244 (16)	0.0309 (18)	0.051 (2)	−0.0019 (14)	0.0074 (16)	0.0084 (17)
C4	0.0296 (17)	0.0229 (16)	0.046 (2)	−0.0061 (13)	−0.0032 (15)	0.0010 (15)
C5	0.0320 (17)	0.0198 (15)	0.0352 (18)	−0.0051 (13)	0.0011 (15)	−0.0021 (14)
C6	0.0300 (16)	0.0153 (14)	0.0287 (17)	−0.0035 (12)	0.0099 (14)	0.0041 (13)
C7	0.0198 (13)	0.0148 (13)	0.0195 (14)	−0.0012 (11)	0.0070 (12)	0.0034 (12)
C8	0.0304 (16)	0.0172 (14)	0.0230 (15)	−0.0008 (12)	0.0092 (13)	−0.0012 (12)
C11	0.0227 (14)	0.0204 (14)	0.0174 (15)	0.0000 (12)	0.0089 (12)	0.0005 (12)
C12	0.0308 (16)	0.0215 (16)	0.0295 (17)	0.0026 (13)	0.0055 (14)	−0.0005 (13)
C13	0.0229 (15)	0.0320 (18)	0.0317 (18)	0.0051 (13)	0.0002 (14)	0.0099 (15)
C14	0.0227 (15)	0.0432 (19)	0.0189 (16)	−0.0106 (14)	0.0048 (13)	−0.0035 (14)
C15	0.0302 (17)	0.0278 (16)	0.0367 (18)	−0.0052 (14)	0.0165 (15)	−0.0104 (14)
C16	0.0230 (15)	0.0246 (15)	0.0247 (16)	0.0009 (12)	0.0096 (13)	0.0002 (13)
C21	0.0228 (14)	0.0133 (13)	0.0168 (14)	0.0046 (11)	0.0064 (12)	0.0036 (11)
C22	0.0230 (15)	0.0422 (18)	0.0228 (16)	0.0007 (14)	0.0092 (13)	−0.0043 (14)
C23	0.0233 (15)	0.046 (2)	0.0361 (19)	−0.0021 (14)	0.0164 (15)	−0.0045 (16)
C24	0.0366 (18)	0.0369 (18)	0.0221 (16)	0.0089 (14)	0.0172 (14)	0.0044 (14)
C25	0.0327 (16)	0.0287 (16)	0.0188 (15)	0.0015 (13)	0.0105 (13)	−0.0016 (13)
C26	0.0227 (14)	0.0209 (15)	0.0243 (15)	−0.0019 (12)	0.0075 (13)	0.0037 (12)
C31	0.0180 (13)	0.0235 (15)	0.0163 (14)	−0.0029 (11)	0.0062 (11)	−0.0012 (12)
C32	0.0285 (16)	0.0309 (16)	0.0251 (16)	0.0050 (13)	0.0155 (13)	0.0039 (14)
C33	0.0423 (19)	0.0336 (19)	0.0308 (18)	0.0146 (15)	0.0146 (16)	0.0066 (15)
C34	0.0257 (16)	0.0344 (18)	0.0354 (18)	0.0106 (14)	0.0111 (15)	−0.0006 (15)
C35	0.0275 (16)	0.0393 (18)	0.0275 (17)	−0.0037 (14)	0.0186 (14)	−0.0066 (14)
C36	0.0263 (15)	0.0240 (15)	0.0212 (15)	−0.0014 (13)	0.0083 (13)	−0.0003 (12)
C41	0.0279 (16)	0.0262 (16)	0.0183 (15)	0.0052 (13)	0.0091 (13)	−0.0026 (13)
C42	0.0324 (18)	0.0322 (17)	0.0352 (19)	−0.0002 (14)	0.0084 (15)	0.0036 (15)
C43	0.0320 (19)	0.043 (2)	0.048 (2)	−0.0010 (16)	0.0056 (17)	−0.0078 (18)
C44	0.0309 (18)	0.063 (3)	0.0275 (18)	0.0101 (18)	0.0021 (15)	−0.0063 (18)
C45	0.042 (2)	0.047 (2)	0.0294 (19)	0.0180 (17)	0.0163 (16)	0.0148 (16)
C46	0.0351 (17)	0.0312 (17)	0.0313 (17)	0.0045 (14)	0.0191 (15)	0.0036 (14)
C51	0.0268 (15)	0.0213 (15)	0.0228 (15)	−0.0110 (12)	0.0074 (13)	−0.0006 (13)
C52	0.0326 (17)	0.0398 (19)	0.0291 (18)	−0.0058 (15)	0.0068 (15)	0.0079 (15)
C53	0.0334 (19)	0.064 (3)	0.031 (2)	−0.0087 (18)	0.0054 (16)	0.0215 (18)
C54	0.044 (2)	0.075 (3)	0.0207 (18)	−0.032 (2)	0.0151 (16)	−0.0044 (19)
C55	0.048 (2)	0.0394 (19)	0.0347 (19)	−0.0236 (17)	0.0243 (16)	−0.0152 (17)
C56	0.0425 (18)	0.0241 (16)	0.0332 (18)	−0.0085 (15)	0.0190 (15)	−0.0007 (15)
C61	0.0297 (16)	0.0177 (15)	0.0244 (15)	0.0024 (12)	0.0099 (13)	0.0055 (12)
C62	0.0382 (18)	0.0236 (15)	0.0352 (18)	−0.0038 (14)	0.0237 (15)	−0.0040 (14)
C63	0.0426 (19)	0.0266 (17)	0.046 (2)	−0.0096 (14)	0.0240 (17)	−0.0114 (15)
C64	0.0348 (18)	0.0287 (17)	0.046 (2)	−0.0032 (14)	0.0246 (16)	−0.0011 (16)
C65	0.0390 (19)	0.0361 (18)	0.0308 (18)	0.0025 (15)	0.0230 (16)	0.0057 (15)
C66	0.0326 (17)	0.0236 (16)	0.0257 (16)	0.0047 (13)	0.0098 (14)	0.0019 (13)

### Geometric parameters (Å, °)

**Table:**

S1—C1	1.779 (3)	C31—C36	1.397 (4)
S1—C7	1.896 (3)	C32—C33	1.381 (4)
N1—C6	1.391 (3)	C32—H32A	0.9500
N1—C8	1.482 (4)	C33—C34	1.362 (4)
N1—H1	0.81 (3)	C33—H33A	0.9500
C1—C2	1.386 (4)	C34—C35	1.378 (4)
C1—C6	1.406 (4)	C34—H34A	0.9500
C2—C3	1.389 (4)	C35—C36	1.381 (4)
C2—H2A	0.9500	C35—H35A	0.9500
C3—C4	1.371 (5)	C36—H36A	0.9500
C3—H3A	0.9500	C41—C42	1.387 (4)
C4—C5	1.371 (4)	C41—C46	1.390 (4)
C4—H4A	0.9500	C42—C43	1.382 (4)
C5—C6	1.397 (4)	C42—H42A	0.9500
C5—H5A	0.9500	C43—C44	1.389 (5)
C7—C21	1.532 (4)	C43—H43A	0.9500
C7—C31	1.541 (4)	C44—C45	1.366 (5)
C7—C11	1.543 (4)	C44—H44A	0.9500
C8—C51	1.534 (4)	C45—C46	1.387 (4)
C8—C41	1.546 (4)	C45—H45A	0.9500
C8—C61	1.548 (4)	C46—H46A	0.9500
C11—C16	1.389 (4)	C51—C52	1.389 (4)
C11—C12	1.390 (4)	C51—C56	1.395 (4)
C12—C13	1.389 (4)	C52—C53	1.385 (5)
C12—H12A	0.9500	C52—H52A	0.9500
C13—C14	1.363 (4)	C53—C54	1.382 (5)
C13—H13A	0.9500	C53—H53A	0.9500
C14—C15	1.380 (4)	C54—C55	1.370 (5)
C14—H14A	0.9500	C54—H54A	0.9500
C15—C16	1.392 (4)	C55—C56	1.383 (4)
C15—H15A	0.9500	C55—H55A	0.9500
C16—H16A	0.9500	C56—H56A	0.9500
C21—C22	1.383 (4)	C61—C66	1.385 (4)
C21—C26	1.399 (4)	C61—C62	1.393 (4)
C22—C23	1.393 (4)	C62—C63	1.396 (4)
C22—H22A	0.9500	C62—H62A	0.9500
C23—C24	1.367 (4)	C63—C64	1.365 (4)
C23—H23A	0.9500	C63—H63A	0.9500
C24—C25	1.383 (4)	C64—C65	1.387 (4)
C24—H24A	0.9500	C64—H64A	0.9500
C25—C26	1.378 (4)	C65—C66	1.380 (4)
C25—H25A	0.9500	C65—H65A	0.9500
C26—H26A	0.9500	C66—H66A	0.9500
C31—C32	1.394 (4)		
			
C1—S1—C7	106.66 (12)	C32—C31—C7	121.7 (2)
C6—N1—C8	127.3 (2)	C36—C31—C7	120.7 (2)
C6—N1—H1	111 (2)	C33—C32—C31	120.6 (3)
C8—N1—H1	115 (2)	C33—C32—H32A	119.7
C2—C1—C6	120.9 (3)	C31—C32—H32A	119.7
C2—C1—S1	120.1 (2)	C34—C33—C32	121.2 (3)
C6—C1—S1	119.0 (2)	C34—C33—H33A	119.4
C1—C2—C3	120.4 (3)	C32—C33—H33A	119.4
C1—C2—H2A	119.8	C33—C34—C35	119.5 (3)
C3—C2—H2A	119.8	C33—C34—H34A	120.3
C4—C3—C2	118.9 (3)	C35—C34—H34A	120.3
C4—C3—H3A	120.6	C34—C35—C36	120.1 (3)
C2—C3—H3A	120.6	C34—C35—H35A	119.9
C5—C4—C3	121.3 (3)	C36—C35—H35A	119.9
C5—C4—H4A	119.3	C35—C36—C31	121.2 (3)
C3—C4—H4A	119.3	C35—C36—H36A	119.4
C4—C5—C6	121.3 (3)	C31—C36—H36A	119.4
C4—C5—H5A	119.3	C42—C41—C46	118.5 (3)
C6—C5—H5A	119.3	C42—C41—C8	118.9 (3)
N1—C6—C5	122.9 (3)	C46—C41—C8	122.5 (3)
N1—C6—C1	120.0 (2)	C43—C42—C41	120.9 (3)
C5—C6—C1	117.1 (3)	C43—C42—H42A	119.5
C21—C7—C31	112.2 (2)	C41—C42—H42A	119.5
C21—C7—C11	111.8 (2)	C42—C43—C44	120.0 (3)
C31—C7—C11	110.0 (2)	C42—C43—H43A	120.0
C21—C7—S1	111.69 (18)	C44—C43—H43A	120.0
C31—C7—S1	108.55 (18)	C45—C44—C43	119.4 (3)
C11—C7—S1	102.17 (16)	C45—C44—H44A	120.3
N1—C8—C51	110.1 (2)	C43—C44—H44A	120.3
N1—C8—C41	105.2 (2)	C44—C45—C46	120.9 (3)
C51—C8—C41	108.7 (2)	C44—C45—H45A	119.5
N1—C8—C61	109.3 (2)	C46—C45—H45A	119.5
C51—C8—C61	111.7 (2)	C45—C46—C41	120.2 (3)
C41—C8—C61	111.7 (2)	C45—C46—H46A	119.9
C16—C11—C12	117.9 (2)	C41—C46—H46A	119.9
C16—C11—C7	120.2 (2)	C52—C51—C56	116.7 (3)
C12—C11—C7	121.8 (2)	C52—C51—C8	122.5 (3)
C13—C12—C11	120.9 (3)	C56—C51—C8	120.8 (3)
C13—C12—H12A	119.5	C53—C52—C51	121.1 (3)
C11—C12—H12A	119.5	C53—C52—H52A	119.4
C14—C13—C12	120.8 (3)	C51—C52—H52A	119.4
C14—C13—H13A	119.6	C54—C53—C52	120.7 (3)
C12—C13—H13A	119.6	C54—C53—H53A	119.6
C13—C14—C15	119.2 (3)	C52—C53—H53A	119.6
C13—C14—H14A	120.4	C55—C54—C53	119.1 (3)
C15—C14—H14A	120.4	C55—C54—H54A	120.5
C14—C15—C16	120.6 (3)	C53—C54—H54A	120.5
C14—C15—H15A	119.7	C54—C55—C56	120.0 (3)
C16—C15—H15A	119.7	C54—C55—H55A	120.0
C11—C16—C15	120.6 (3)	C56—C55—H55A	120.0
C11—C16—H16A	119.7	C55—C56—C51	122.1 (3)
C15—C16—H16A	119.7	C55—C56—H56A	118.9
C22—C21—C26	117.1 (3)	C51—C56—H56A	118.9
C22—C21—C7	121.5 (2)	C66—C61—C62	118.3 (3)
C26—C21—C7	121.2 (2)	C66—C61—C8	119.5 (3)
C21—C22—C23	121.6 (3)	C62—C61—C8	122.2 (3)
C21—C22—H22A	119.2	C61—C62—C63	119.9 (3)
C23—C22—H22A	119.2	C61—C62—H62A	120.1
C24—C23—C22	120.4 (3)	C63—C62—H62A	120.1
C24—C23—H23A	119.8	C64—C63—C62	121.2 (3)
C22—C23—H23A	119.8	C64—C63—H63A	119.4
C23—C24—C25	119.1 (3)	C62—C63—H63A	119.4
C23—C24—H24A	120.4	C63—C64—C65	119.2 (3)
C25—C24—H24A	120.4	C63—C64—H64A	120.4
C26—C25—C24	120.6 (3)	C65—C64—H64A	120.4
C26—C25—H25A	119.7	C66—C65—C64	120.1 (3)
C24—C25—H25A	119.7	C66—C65—H65A	119.9
C25—C26—C21	121.2 (3)	C64—C65—H65A	119.9
C25—C26—H26A	119.4	C65—C66—C61	121.3 (3)
C21—C26—H26A	119.4	C65—C66—H66A	119.3
C32—C31—C36	117.4 (3)	C61—C66—H66A	119.3
			
C7—S1—C1—C2	77.1 (3)	C11—C7—C31—C36	68.2 (3)
C7—S1—C1—C6	−105.3 (2)	S1—C7—C31—C36	−42.8 (3)
C6—C1—C2—C3	3.0 (4)	C36—C31—C32—C33	1.6 (4)
S1—C1—C2—C3	−179.4 (2)	C7—C31—C32—C33	176.3 (3)
C1—C2—C3—C4	−0.7 (4)	C31—C32—C33—C34	−0.8 (5)
C2—C3—C4—C5	−1.5 (5)	C32—C33—C34—C35	−0.7 (5)
C3—C4—C5—C6	1.3 (5)	C33—C34—C35—C36	1.3 (5)
C8—N1—C6—C5	31.6 (5)	C34—C35—C36—C31	−0.5 (4)
C8—N1—C6—C1	−151.0 (3)	C32—C31—C36—C35	−0.9 (4)
C4—C5—C6—N1	178.4 (3)	C7—C31—C36—C35	−175.7 (2)
C4—C5—C6—C1	1.0 (4)	N1—C8—C41—C42	51.0 (4)
C2—C1—C6—N1	179.3 (3)	C51—C8—C41—C42	−66.9 (3)
S1—C1—C6—N1	1.7 (3)	C61—C8—C41—C42	169.5 (3)
C2—C1—C6—C5	−3.1 (4)	N1—C8—C41—C46	−130.5 (3)
S1—C1—C6—C5	179.3 (2)	C51—C8—C41—C46	111.6 (3)
C1—S1—C7—C21	52.5 (2)	C61—C8—C41—C46	−12.0 (4)
C1—S1—C7—C31	−71.7 (2)	C46—C41—C42—C43	2.1 (5)
C1—S1—C7—C11	172.12 (18)	C8—C41—C42—C43	−179.3 (3)
C6—N1—C8—C51	−94.4 (3)	C41—C42—C43—C44	−0.4 (5)
C6—N1—C8—C41	148.7 (3)	C42—C43—C44—C45	−1.1 (5)
C6—N1—C8—C61	28.6 (4)	C43—C44—C45—C46	0.9 (5)
C21—C7—C11—C16	−98.2 (3)	C44—C45—C46—C41	0.8 (5)
C31—C7—C11—C16	27.1 (3)	C42—C41—C46—C45	−2.2 (5)
S1—C7—C11—C16	142.2 (2)	C8—C41—C46—C45	179.2 (3)
C21—C7—C11—C12	78.6 (3)	N1—C8—C51—C52	21.6 (4)
C31—C7—C11—C12	−156.0 (3)	C41—C8—C51—C52	136.3 (3)
S1—C7—C11—C12	−40.9 (3)	C61—C8—C51—C52	−100.0 (3)
C16—C11—C12—C13	−2.0 (4)	N1—C8—C51—C56	−159.5 (3)
C7—C11—C12—C13	−179.0 (3)	C41—C8—C51—C56	−44.8 (3)
C11—C12—C13—C14	1.6 (5)	C61—C8—C51—C56	78.8 (3)
C12—C13—C14—C15	−0.4 (5)	C56—C51—C52—C53	−3.9 (4)
C13—C14—C15—C16	−0.5 (5)	C8—C51—C52—C53	175.0 (3)
C12—C11—C16—C15	1.2 (4)	C51—C52—C53—C54	1.3 (5)
C7—C11—C16—C15	178.2 (3)	C52—C53—C54—C55	2.0 (5)
C14—C15—C16—C11	0.0 (4)	C53—C54—C55—C56	−2.3 (5)
C31—C7—C21—C22	−119.3 (3)	C54—C55—C56—C51	−0.5 (4)
C11—C7—C21—C22	4.7 (3)	C52—C51—C56—C55	3.6 (4)
S1—C7—C21—C22	118.5 (2)	C8—C51—C56—C55	−175.3 (3)
C31—C7—C21—C26	55.8 (3)	N1—C8—C61—C66	45.9 (3)
C11—C7—C21—C26	179.9 (2)	C51—C8—C61—C66	168.0 (2)
S1—C7—C21—C26	−66.4 (3)	C41—C8—C61—C66	−70.1 (3)
C26—C21—C22—C23	0.7 (4)	N1—C8—C61—C62	−132.9 (3)
C7—C21—C22—C23	176.0 (3)	C51—C8—C61—C62	−10.8 (4)
C21—C22—C23—C24	0.6 (5)	C41—C8—C61—C62	111.1 (3)
C22—C23—C24—C25	−0.9 (5)	C66—C61—C62—C63	−0.7 (4)
C23—C24—C25—C26	−0.2 (4)	C8—C61—C62—C63	178.1 (3)
C24—C25—C26—C21	1.7 (4)	C61—C62—C63—C64	0.0 (5)
C22—C21—C26—C25	−1.9 (4)	C62—C63—C64—C65	0.1 (5)
C7—C21—C26—C25	−177.2 (2)	C63—C64—C65—C66	0.5 (5)
C21—C7—C31—C32	18.8 (3)	C64—C65—C66—C61	−1.1 (4)
C11—C7—C31—C32	−106.3 (3)	C62—C61—C66—C65	1.2 (4)
S1—C7—C31—C32	142.7 (2)	C8—C61—C66—C65	−177.6 (3)
C21—C7—C31—C36	−166.7 (2)		

### Hydrogen-bond geometry (Å, °)

**Table:**

*D*—H···*A*	*D*—H	H···*A*	*D*···*A*	*D*—H···*A*
N1—H1···S1	0.81 (3)	2.46 (3)	2.984 (3)	123 (1)
